# Liquid chromatography-mass spectrometry approaches for analysis of polychlorinated alkanes: a review

**DOI:** 10.1007/s11356-026-38341-3

**Published:** 2026-10-24

**Authors:** Laura Lazdiņa, Ingus Pērkons

**Affiliations:** 1 Institute of Food Safety, Animal Health and Environment “BIOR”, Lejupes Street 3, Riga, LV-1076 Latvia; 2 University of Latvia, Jelgavas Street 1, Riga, LV-1004 Latvia

**Keywords:** Polychlorinated alkanes, Chlorinated paraffins, Liquid chromatography, Mass spectrometry, Persistent organic pollutants, Adduct formation, Ionization additives, Spectral interferences

## Abstract

Polychlorinated alkanes (PCAs), the primary components of technical mixtures known as chlorinated paraffins (CPs), are complex and persistent pollutants of growing environmental and analytical concern. Their widespread use and toxicological properties, particularly those of short- and medium-chain PCAs (C_10–17_), have prompted increased regulatory and scientific attention. This review provides a comprehensive overview of recent advances in the analysis of PCAs using liquid chromatography coupled with mass spectrometry (LC–MS), with emphasis on separation, ionization, and detection strategies. Key developments in stationary and mobile phase selection, gradient optimization, and the role of mobile phase additives in enhancing ionization are critically evaluated. Particular attention is paid to the challenges posed by the structural complexity of PCAs, including isomeric diversity and mass spectral interferences, and how they are addressed by high-resolution and tandem mass spectrometric techniques. Variability in instrumental response across carbon-chain lengths and chlorination degrees is also examined. The review highlights current methodological advancements and limitations, and outlines perspectives for improving analytical reliability.

**Graphical Abstract:**

**Figure:**
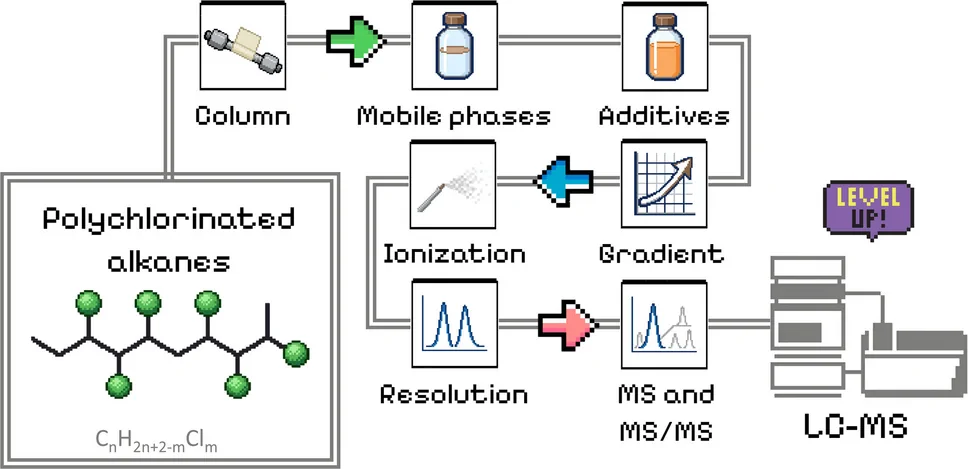

## Introduction

Polychlorinated alkanes (PCAs), the principal components of technical chlorinated paraffin (CP) mixtures, are a complex group of persistent and chemically resistant compounds. The primary applications of PCA mixtures include flame retardants, plasticizers, and lubricants in metalworking (Glüge et al. 2016). These compounds have a general formula of C_x_H_2x+2-y_Cl_y_ and are usually separated into three groups distinguished by their carbon chain length: PCAs-C_10–13_ or short-chain CPs (SCCPs), PCAs-C_14–17_ or medium-chain CPs (MCCPs), and PCAs-C_18–30_ or long-chain CPs (LCCPs). The chlorine content of PCAs is typically estimated to be between 30 and 70% by weight (Vetter et al. 2022; Fernandes et al. 2023). Because terminology in PCA/CP research is not fully harmonized across disciplines, we follow the recommended terms and abbreviations proposed by Fernandes et al. (2023) and refer readers to that paper for an overview of common nomenclature and abbreviations used in the field (Fernandes et al. 2023).

PCAs are persistent and are widely detected in environmental and food matrices (Nevondo and Okonkwo 2021). Their tendency to bioaccumulate and biomagnify through the food chain contributes to elevated levels in biota and food products (van Mourik et al. 2016; Castro et al. 2019), and their widespread presence in different food products (Krätschmer et al. 2021; McGrath et al. 2021a). Due to this, there are risks of long-term exposure and, consequently, potentially adverse effects on the health of various living organisms, including animals and humans (Kobetičová and Černý 2018; Chen et al. 2023).

Studies suggest that long-term exposure to PCAs from the environment or consumer products may pose health risks, including liver and kidney toxicity, endocrine disruption, neurotoxicity, and developmental and reproductive effects (Huang et al. 2023; Chen et al. 2023). In addition, International Agency for Research on Cancer classified chlorinated paraffins with an average carbon chain length of C_12_ and ~ 60% chlorination as Group 2 B (possibly carcinogenic to humans). In 2017, PCAs-C_10–13_ were listed as persistent organic pollutants (POPs) under the Stockholm Convention (UNEP 2017). Longer-chain PCAs nevertheless share several properties of concern with their shorter-chain counterparts, including bioaccumulation and biomagnification. In 2025, PCAs-C_14–17_ were also included in Annex A according to the Stockholm Convention (POPRC-20/2), though with multiple exceptions (UNEP 2024; Council of the European Union 2025). Potentially, these restrictions could cause a higher reliance on PCAs with even longer carbon chains (i.e., C ≥ 18). For instance, Guida et al. (2023) analyzed more than 30 technical mixtures produced between the 1970 s and 2022, showing that these mixtures are highly diverse and that regulatory measures have already driven a gradual shift from shorter- to longer-chain PCAs (Guida et al. 2023). As there is currently limited scientific evidence on the adverse health effects of PCAs with very long carbon chains (C ≥ 18), it is undecided whether further regulations are necessary for these PCAs (van Mourik et al. 2016; Glüge et al. 2018; Zellmer et al. 2020).

Over time, various methods for analyzing PCAs in different matrices have been developed, including gas and liquid chromatography, such as direct liquid injection mass spectrometry without a chromatographic column (Bogdal et al. 2015), super-critical fluid chromatography (Tomasko et al. 2023), and even planar chromatography (Hanari and Nakano 2022). However, the demand for liquid chromatography-based methods has increased substantially in recent years. This development is closely tied to the regulatory restrictions on short-chain PCAs, which have changed the composition of commercial CP mixtures. As manufacturers shifted production toward longer-chain PCAs, analytical challenges emerged, since these congeners are less volatile and therefore cannot be characterized efficiently by conventional GC-based methods. Consequently, liquid chromatography-based methods have recently been at the forefront of many research studies (Mézière et al. 2020b). This review provides a comprehensive overview of liquid chromatography-mass spectrometry (LC–MS) approaches applied to PCA analysis, summarizing recent advances in stationary and mobile phase selection, ionization strategies, and mass analyzer performance, as well as the key findings these methods have enabled across environmental, food, and product-related studies.

## Chromatographic separation of PCAs

PCAs, depending on the chain length and chlorine content, can have hundreds to thousands of isomers per homologue group. For PCAs-C_10–13_, with the premise that there is only one Cl atom on each carbon and the chlorination degree is limited to 5–10 Cl atoms per molecule, over 6000 different isomers have been calculated (Vetter et al. 2022). However, the number is expected to be higher due to the possibility of more than 1 chlorine per carbon and a higher or lower chlorination degree. More detailed information on the structural diversity and complexity of PCAs, including historical perspectives and isomer calculations, is provided by Vetter et al. (2022). The varying physical and chemical properties can also significantly affect separation efficiency. The extreme complexity of PCA mixtures currently makes the complete separation of all homologue groups impossible. Hence, several recent studies have explored multiple prospective stationary and mobile phase combinations in liquid chromatography to improve separation of PCAs (Mézière et al. 2020b; Amoura et al. 2024). To illustrate the diversity of LC methods reported for PCA analysis, Table 1 summarizes key chromatographic parameters from representative studies in the literature. The following subsections discuss these and other relevant studies in more detail.

**Table 1:** Reported chromatographic conditions for LC–MS analysis of PCAs, including column type, mobile phases, gradient range, and run time

PCA homologue groups analyzed (Reference)	Column (dimensions)	Mobile phases	Organic phase min–max % (flowrate)	Gradient length, min
PCAs-C_6–36_ (Macan Schönleben et al. 2026); PCAs-C_10–36_ (McGrath et al. 2022a); PCAs-C_10–25_ (McGrath et al. b)	Zorbax Eclipse Plus C18 RRHD (50 × 2.1 mm, 1.8 μm)	A: water; B: 25 μM NH_4_Cl in MeOH	10%−95% B (0.2 mL/min)	12–14 min
PCAs-C_10–17_ (Perkons et al. 2025)	Kinetex Phenyl-Hexyl (100 × 2.1 mm, 2.6 µm)	A: 5 mM NH_4_CH_3_COO in water; B: 5 mM NH_4_CH_3_COO in MeOH	50%−100% B (0.25 mL/min)	22 min
PCAs-C_10–13_ (Matsukami et al. 2020); PCAs-C_10–20_ (Guida et al. 2023)	Zorbax SB-CN RRHD (50 × 2.1 mm, 1.8 μm)	A: 5 mM NH_4_CH_3_COO in water; B: 5 mM NH_4_CH_3_COO in MeOH	60%−99% B (0.4 mL/min)	20.1 min
PCAs-C_9–34_ (Knobloch et al. 2024; Mendo Diaz et al. 2024); PCAs-C_9–30_ (Mendo Diaz et al. 2025)	Zorbax SB C18-RRHD (50 × 3 mm, 1.8 μm)	A: water; B: MeOH/DCM (9:1, v/v)	40%−98% B (0.4 mL/min)	25 min
PCAs-C_8–36_ (Mézière et al. 2020a)	Hypersil Gold C18 (100 × 2.1 mm, 1.9 µm)	A: water; B: ACN	70% B-100% B (0.4 mL/min)	12 min
PCAs-C_8–17_ (Wu et al. 2019)	Hypersil Gold C18 (100 × 2.1 mm, 1.9 µm)	A: water with 0.02% acetic acid and 5 mM NH_4_CH_3_COO; B: MeOH; C: ACN	70%−100% B/C (0.3 mL/min)	25 min
PCAs-C_6–25_ (Sun et al. 2022)	Poroshell 120 ECC8 (100 × 2.1 mm, 2.7 μm)	A: water; B: 10 µM Ph_4_PCl in MeOH	10%−100% B (0.1 mL/min)	13 min
PCAs-C_10–20_ (Tomasko et al. 2023)	Acquity UPLC BEH C18 (100 × 2.1 mm, 1.7 µm)	A: MeOH; B: iPr/MeOH/water (65:30:5, v/v/v)	10%−100% B (0.2 mL/min)	11 min
PCAs-C_10–26_ (Huang et al. 2021)	Accucore C18 (2.1 × 100 mm, 2.6 μm)	A: 50 µM (CH_3_)_4_NCl in water; B: 50 µM (CH_3_)_4_NCl in ACN	30%−100% B (0.3 mL/min)	14 min
PCAs-C_10–35_ (Cariou et al. 2016)	Hypersil Gold C18 (100 × 2.1 mm, 1.9 μm)	A: 20 mM NH_4_CH_3_COO in water; B: 20 mM NH_4_CH_3_COO in MeOH/ACN(50:50, v/v)	5%−100% B (0.4 mL/min)	29 min
PCAs-C_10–25_ (Zheng et al. 2019); PCAs-C_10–17_ (Shen et al. 2023)	Agilent Eclipse Plus C18 (50 × 2.1 mm, 1.8 μm)	A: water; B: 50 μM NH_4_Cl in MeOH	5%−100% B (0.2 mL/min)	20 min
PCAs-C_10–17_ (Zhou et al. 2020)	Poroshell 120 PFP (100 × 2.1 mm, 2.7 μm)	A: 10 mM NH_4_CH_3_COO in water; B: MeOH	40%−100% B (0.25 mL/min)	13 min

### Stationary phases

Due to PCA hydrophobic properties and interactions, octadecylsilane (C18) type columns have commonly been selected to separate PCA homologues (Amoura et al. 2024). Recently, it was highlighted that some groups might exhibit hydrogen donor properties, suggesting that a broader range of stationary phases could improve the separation of PCAs (Hammer et al. 2021).

As seen in Table 1, several studies have evaluated alternative stationary phases beyond C18 for the analysis of PCAs. For instance, Zhou et al. (2020) used a pentafluorophenyl (PFP) stationary phase to analyze PCAs-C_10–17_ in wine. PFP is said to be more efficient at separating halogenated compounds than conventional aliphatic phases due to an enhanced dipole and π-π interactions (Santasania and Bell 2016; Amoura et al. 2024). However, the results of Zhou et al. (2020) and Amoura et al. (2024) showed that, despite the potential for additional interactions, the PFP column did not provide a significant improvement in PCA separation compared to the conventional C18 phase. For analysis of samples from a municipal waste incinerator, Matsukami et al. (2019) used a Phenyl-Hexyl column, which, in addition to hydrophobic interactions, offers π-π based interactions and possible hydrogen-bonding-based interactions (Croes et al. 2005). The authors reported that the analysis of the PCAs-C_14–17_ homologues was successful, although separation from other PCA homologues, which could cause interference with PCAs-C_14–17_, was not investigated. In another study by Matsukami et al. (2020) C18 and cyanopropylsilane (CN) type columns were compared in the separation of PCAs-C_10–13_ homologues. Chromatograms provided in their work illustrate a clear improvement in peak shape and separation on the CN phase. For example, the resolution between PCAs-C_10_Cl_6_ and PCAs-C_13_Cl_6_ was slightly below 1 on the C18 column (broad peaks, 2.6–2.8 min wide), whereas on the CN column the same homologues were baseline separated with a resolution close to 2 (sharp peaks, 0.8–0.9 min wide). These results demonstrate that, at least in this study, the CN column provided markedly better performance than C18. CN-type columns contain a lipophilic propyl spacer that allows for hydrophobic interactions, but their cyanopropyl groups also introduce polar selectivity. As a result, retention can involve not only hydrophobic forces but also dipole–dipole and, in some cases, π–π interactions. Previous studies have shown that dipole–dipole interactions are particularly important for compounds with strong dipole moments (Croes et al. 2005; Kouskoura et al. 2014). In the case of PCAs, the presence of multiple chlorine substituents creates localized polar regions and molecular dipole moments, which may enhance retention on CN-type phases compared with purely hydrophobic columns. After Matsukami et al. (2020) reported these findings, other studies have also used CN-type columns (McGrath et al. 2021a; Guida et al. 2022; Tran et al. 2025).

Recently, Amoura et al. (2024) published a comprehensive study comparing the influence of 6 different stationary phases (C18, triacontyl (C30), Hypercarb, PFP, Phenyl-Hexyl, CN) on PCA separation and response. Hypercarb and CN were excluded after preliminary tests showed incomplete homologue patterns, with highly chlorinated homologues not detected. However, the study did not discuss in detail why these homologues were not observed. For the remaining phases, retention increased with chain length. For PCAs-C_10–13_ retention also increased along with chlorination degree for all columns. However, on C18 and C30 phases higher-chain PCAs (C ≥ 14) showed non-monotonic behavior: low-chlorinated homologues (Cl_4_-Cl_5_) eluted after medium-chlorinated ones (Cl_7_–Cl_8_), with retention increasing again only above Cl_8_. One possible explanation is changes in the shape of longer-chain PCAs, which may lower the accessible hydrophobic surface area and weaken interactions with C18/C30 phases. In addition, lower-chlorinated higher-chain PCAs retain longer uninterrupted -(CH_2_)- segments, which may enhance interactions with the hydrophobic stationary phase compared with more highly chlorinated analogues of the same carbon length. In contrast, on the Phenyl-Hexyl column retention within each chain length generally followed chlorine content, with higher chlorination leading to longer retention. On the PFP column, the retention of longer-chain homologues (C ≥ 18) was dictated almost exclusively by chain length, with homologues of the same carbon number but different degrees of chlorination eluting at nearly the same time.

**Fig. 1:**
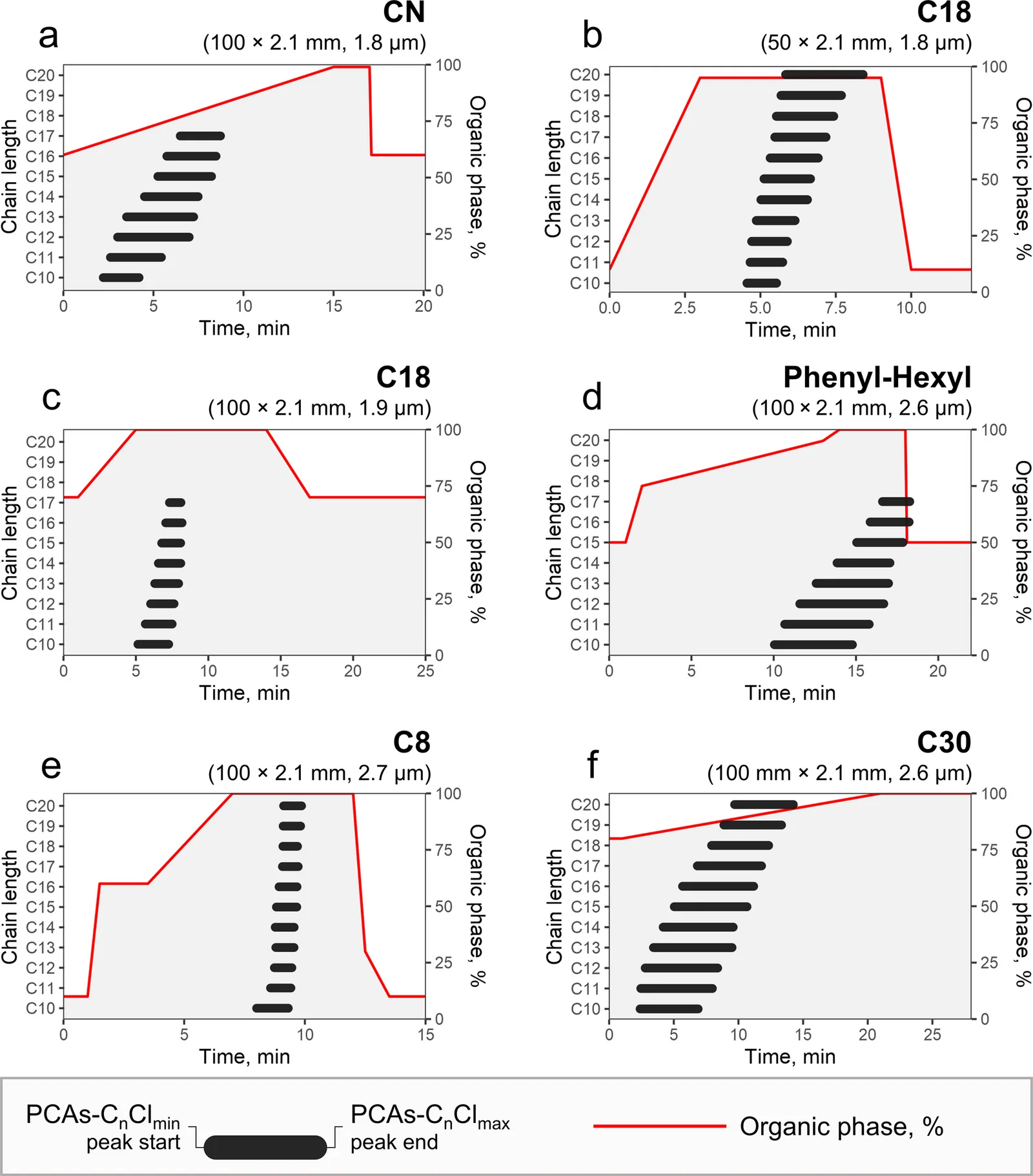
Comparison of literature reported retention time ranges of PCA homologue groups (C_10_-C_20_) and gradient programs across five LC stationary phases. **a**–**f** The literature sources as follows: **a** Guida et al. (2023); **b** McGrath et al. (2022b); **c** Wu et al. (2019); **d** Perkons et al. (2025); **e** Fang et al. (2025); and **f** Amoura et al. (2024)

To visualize the overall chromatographic behavior of PCAs, Fig. 1 compares reported retention time ranges of PCA homologue groups (C_10_-C_20_) and the corresponding gradient programs across six published LC methods employing different stationary phases. Based on the literature data summarized here, CN, Phenyl-Hexyl, and C30 columns appear to offer the most promising separation of PCA chain-length homologues. Nevertheless, the overlap between chain-length homologue groups remains significant. The comparison also shows that the gradient program plays a crucial role in the achieved separation, as homologue elution tends to start within a relatively narrow organic phase composition window. The influence of gradient setting is discussed in more detail in the next section.

Beyond carbon chain length and total chlorine number, the chlorine substitution pattern can influence both LogK_ow_ and LC retention. Experimental and modelling work shows that specific structural differences, such as, vicinal vs geminal chlorination and the length of unsubstituted methylene segments, can shift LogK_ow_ (Bettina et al. 2011; Endo and Hammer 2020). In general, congeners with longer uninterrupted -(CH_2_)- segments and more clustered chlorination appear more hydrophobic, whereas more evenly distributed substitution may reduce hydrophobic interactions and decrease retention on non-polar phases.

**Fig. 2:**
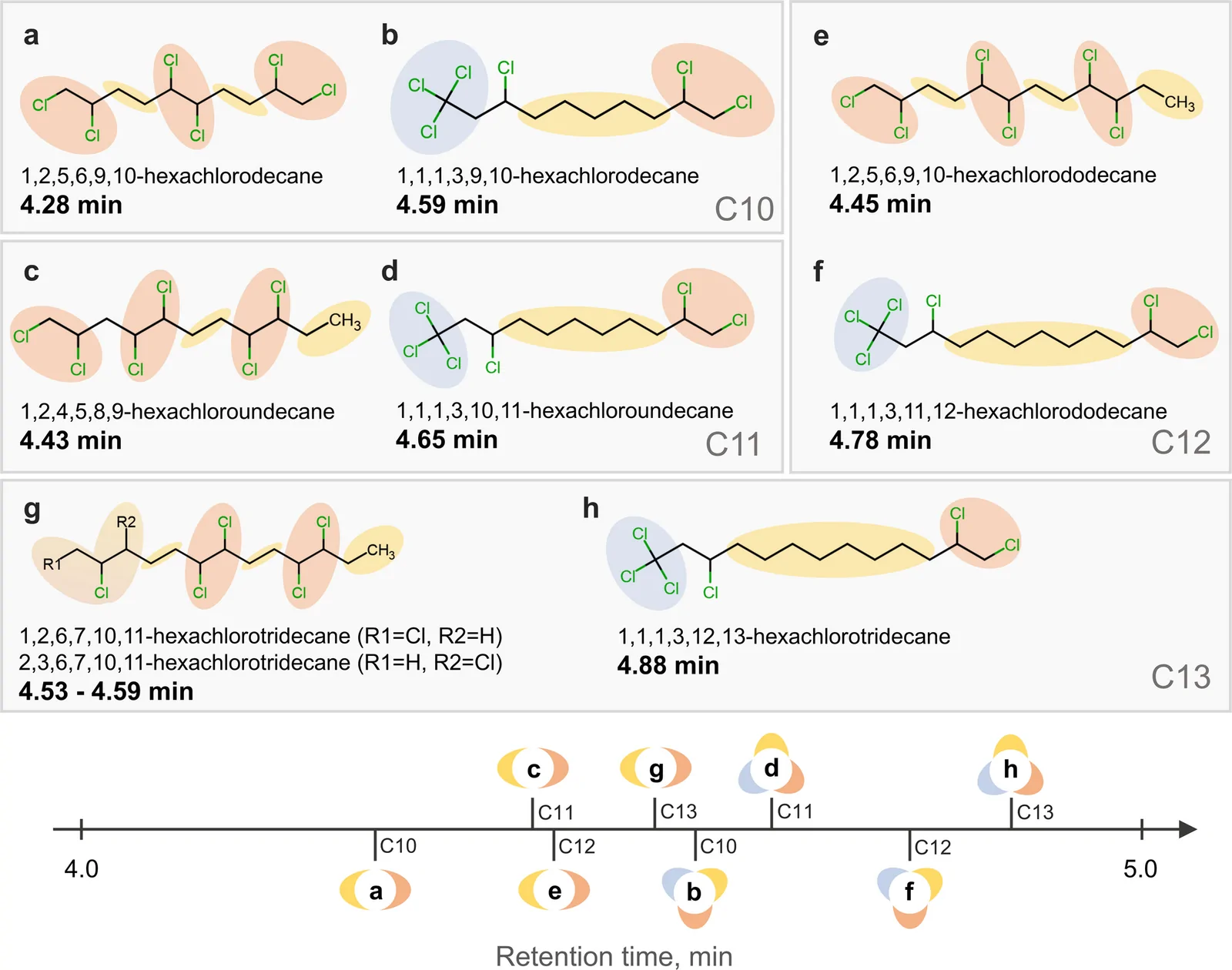
Effect of chlorine substitution pattern on the retention of C_10_-C_13_Cl_6_ PCA congeners on a C18 column. Retention times of the individual peaks were derived from chromatograms reported by Ricci et al. (2024). Blue indicates geminal chlorination, orange indicates vicinal chlorination, and yellow highlights unsubstituted regions of the carbon skeleton

A practical example of the structural difference effect can be observed in a study by Ricci et al. (2024), where the authors analyzed different C_10_-C_13_Cl_6_ congeners on a C18 column. In Fig. 2, structures of these congeners are shown, with highlighted intramolecular structural variations that may cause retention differences, while the lower part of the figure shows the retention time of each congener along the x-axis. The retention time data shown in the figure were obtained by examining the chromatograms provided in the cited article. It could be observed that congeners with geminal Cl atoms showed higher retention times in comparison to congeners with a more even distribution of Cl atoms. This is consistent with the broad, hump-like signals typically observed for samples containing PCA technical mixtures, where many isomers share the same chain length and chlorination degree but differ in chlorine substitution pattern and therefore elute across a broader retention time window.

### Mobile-phase composition and gradient conditions

Mobile phase composition and gradient strength are key determinants of retention and resolution in RP-LC analysis of complex PCA mixtures. Most methods use aqueous acetonitrile or aqueous methanol as the mobile phase (Amoura et al. 2024). Less frequently, ternary water–methanol–acetonitrile systems have been reported (Wu et al. 2019, 2022), as well as more complex eluents such as water (A) and isopropanol–methanol–water (65:30:5, v/v/v; B) (Tomasko et al. 2023).

Against this background, Amoura et al. (2024) systematically compared H_2_O/ACN and H_2_O/MeOH under chlorine-enhanced conditions using post-column addition of an ACN/DCM mixture (50:50, v/v). In both cases, chloride adducts ([M + Cl]^−^) were the dominant ions, while deprotonated ions ([M-H]^−^) were also consistently observed. When expressing the [M-H]^−^ signal as a fraction of the combined ([M-H]^−^  + [M + Cl]^−^) intensity, the relative contribution of [M-H]^−^ was slightly higher with H_2_O/MeOH. In contrast, ACN favored formation of [M + Cl]^−^ and thereby simplifying spectra. However, even in the most extreme cases, the [M-H]^−^ ion contributed no more than about 30% of the total signal with MeOH and no more than about 10% with ACN. This effect was most pronounced for lower-chain PCAs with a high degree of chlorination. Meanwhile, in terms of absolute signal intensity, MeOH increased PCA responses by approximately 1.5-fold compared with ACN. A similar enhancement in response with MeOH has also been reported by Huang et al. (2021). Wu et al. (2019) showed that compared to MeOH, ACN tends to suppress the sensitivity of PCAs with lower chlorine contents, especially for Cl_5_ congeners. However, they still introduced ACN later in the gradient after PCA elution to flush matrix components from the column and maintain column performance. Furthermore, the effect of ACN on chromatographic retention should be also considered. Independent of adduct formation, ACN’s nitrile group can engage in π–π interactions with aromatic systems, which may reduce analyte-stationary phase interactions (Marchand et al. 2005; Yang et al. 2005). On phenyl-type columns, this could diminish one of the retention mechanisms. Methanol has been reported to enhance π–π interactions on phenyl phases in some cases, which may make it a more suitable mobile-phase organic modifier for such stationary phases (Marchand et al. 2005; Yang et al. 2005). Overall, both ACN and MeOH can be applied successfully, but their suitability depends on the analytical priority. MeOH appears more suitable when maximizing signal intensity is the main goal and sufficient selectivity can be ensured by HRMS. By contrast, ACN may be the better choice when reducing spectral complexity is more critical, particularly in methods where resolution is limiting.

Almost all of the reviewed studies employed gradient elution with a binary mobile-phase program. The main differences between most methods were the starting composition of the organic phase. Some studies relied on gradients with a relatively low initial percentage (around 10%) while others started higher (50–70%) to promote earlier elution of PCAs. Most methods ramped the organic phase to 98–100% by the end of the gradient to ensure full elution of PCAs as well as matrix components from the column. For example, McGrath et al. (2022a) used methanol as mobile phase B with a final concentration of 95%, whereas Mézière et al. (2020a) used acetonitrile with the gradient reaching 100%. Overall, reaching 100% organic phase, or at least coming very close to it, also appears to be important when longer-chain PCAs are included in the analysis. As shown in Fig. 1b, a final organic-phase content of 95% on a C18 column resulted in elution of PCAs-C_20_ over a relatively broad time window compared with lower-chain PCAs, indicating that elution of the least polar PCAs would require higher solvent strength. In addition, sample extracts may contain other highly non-polar components that need to be washed from the column to help maintain long-term column performance and longevity.

Gradient steepness also varied: Yuan et al. (2021) used a fast UPLC program with short ramp and hold times, achieving a total run time of about 6 min for PCAs-C_6–30_ on Orbitrap MS operated at a resolving power of 120,000 FWHM at m/z 200, whereas Matsukami et al. (2020) employed a more gradual program with a 25-min run time for PCAs-C_10–13_ by LC–MS/MS. In general, fast LC gradients are suitable when extensive chromatographic resolution is not required, for example when HRMS can separate PCAs from common interferences. In contrast, longer and more carefully optimized gradients are advantageous when MS resolving power is lower (e.g., bench-top TOF instruments) or when using MS/MS to minimize isobaric and matrix interferences. Among the reviewed methods, CN, Phenyl-Hexyl, and C30 columns combined with carefully optimized gradients appear capable of separating at least some critical homologue pairs that form isobaric MS signals under low-resolution MS setting (mostly C_n_Cl_m_ and C_n+5_Cl_m-2_ groups). Yet, systematic data are not available for every potentially interfering pair. Furthermore, as illustrated in Fig. 1, the onset of PCA elution across the different studies occurs within a relatively narrow organic-phase composition window. This indicates that PCA retention is highly sensitive to solvent strength and that even modest differences in gradient profile can substantially influence the separation of chain-length homologue groups. In general, steeper gradients (Fig. 1b, c, and e) were associated with a greater degree of co-elution, whereas a more gradual increase in organic-phase content (Fig. 1a, d, and f) resulted in a broader distribution of elution windows and, consequently, slightly improved homologue separation. However, this improvement in homologue separation comes at the cost of sensitivity, as the analyte signal is distributed over broader chromatographic humps. This trade-off should therefore be considered when high method sensitivity is required.

## Ionization of PCAs in LC–MS

Ionization is an integral part of PCA analysis, as it enables efficient detection and identification of homologues by mass spectrometry. The efficiency of PCA ionization depends not only on the molecular structure and physicochemical properties of the homologues but also on experimental conditions such as the choice of solvent, additives, and ion source parameters. The first reported LC–MS application for PCA analysis was presented by Zencak and Oehme (2004), who used chloride-enhanced atmospheric-pressure chemical ionization to promote the formation of [M + Cl]^−^ adducts. Since then, several LC–MS ionization strategies have been developed for different analytical purposes (Bogdal et al. 2015; Mézière et al. 2020b; Amoura et al. 2024). Table 2 summarizes selected ionization- and MS-related parameters from representative studies, including the additives used, the measured adducts, the ionization source, and the mass spectrometric instrumentation. All mass resolving power values reported in the table are defined using the full width at half maximum (FWHM). The following subsections discuss these examples together with additional relevant studies in more detail.

**Table 2:** Additives used in LC–MS analysis of PCAs to promote adduct formation, grouped by mode of introduction, ionization source, detected adduct, and instrument type

Additive	Ionization source	Adduct	Instrument (resolution)	References
**Mobile phase additive introduction**				
25 μM NH_4_Cl	ESI	[M + Cl]^−^	Q-ToF–MS (40,000)	(McGrath et al. 2022b, 2022a; Macan Schönleben et al. 2026)
25 μM NH_4_Cl	ESI	[M + Cl]^−^	Fourier transformation ion cyclotron resonance MS (120,000)	(Perkons et al. 2022)
50 μM NH_4_Cl	ESI	[M + Cl]^−^	Q-ToF–MS (20,000); Orbitrap MS (70,000)	(Zheng et al. 2019; Shen et al. 2023)
MeOH/DCM (9:1) as mobile phase B	APCI	[M + Cl]^−^	Orbitrap MS (120,000 − 140,000)	(Knobloch et al. 2024; Mendo Diaz et al. 2024, 2025)
10 µM Ph_4_PCl	ESI	[M + Cl]^−^	Orbitrap MS (140,000)	(Sun et al. 2022)
50 µM ((CH_3_)_4_NCl)	ESI	[M + Cl]^−^	Orbitrap MS (70,000)	(Huang et al. 2021)
5 mM NH_4_CH_3_COO	ESI	[M + CH_3_COOH-H]^−^	Triple quadrupole MS/MS (low-resolution)	(Matsukami et al. 2020; Guida et al. 2023; Perkons et al. 2025)
0.02% CH_3_COOH and 5 mM NH_4_CH_3_COO	ESI	[M-H]^−^	Orbitrap MS (120,000)	(Wu et al. 2019)
20 mM NH_4_CH_3_COO	ESI	[M + CH_3_COOH-H]^−^;[M-H]^−^	Orbitrap MS (70,000)	(Cariou et al. 2016)
**Post-column introduction of the additive**				
ACN/DCM (1:1, v/v)	ESI	[M + Cl]^−^	Orbitrap MS (140,000)	(Mézière et al. 2020a)
DCM	APCI	[M + Cl]^−^	Q-ToF–MS (10,000)	(Bogdal et al. 2015)
HCBr_3_	APCI	[M + Br]^−^	Q-ToF–MS (10,000)	(Yuan et al. 2018)
HCCl_3_	APCI	[M + Cl]^−^	Ion trap MS (low-resolution)	(Zencak and Oehme 2004)
**Other modes of additive introduction**				
Pre-injection addition of CHBr_3_ directly to the sample	ESI	[M + Br]^−^	Q-ToF–MS (40,000)	(Parvizian et al. 2024)

### Ionization sources used in LC–MS analysis of PCAs

When liquid chromatography is applied to PCA analysis, ionization is typically achieved with either electrospray ionization (ESI) or atmospheric-pressure chemical ionization (APCI). ESI and APCI are considered “soft ionization” techniques that allow the generation of pseudo-molecular ions with less in-source fragmentation and formation of interfering transformation products. This is crucial considering the already complex nature of PCAs and their mass spectra (Yuan et al. 2016; Schinkel et al. 2017).

Despite slight differences, both methods have been used widely for the analysis of PCAs. In an interlaboratory study reported by Hanari et al. (2025), a common reference material (a technical mixture) was measured using various instrumental setups including LC-Orbitrap MS with both ESI and APCI ionization sources. Comparison of relative profiles of PCAs-C_10–13_ homologue profiles showed that both ionization sources produced a close match. Similarly, Schinkel et al. (2018a) compared homologue profiles of a mixture of PCAs-C_13_ with three methods: two APCI-MS and one ESI–MS. The results showed good agreement across ionization sources. In contrast, Krätschmer and Schächtele (2019) demonstrated that there can be a significant mismatch of homologue profiles across techniques in fortified food samples, even when laboratories used nominally the same approach. Even with the same instrumental setup (direct-injection APCI-TOF–MS), substantial differences arose between laboratories. It should also be noted that the study by Krätschmer and Schächtele (2019) was based on fortified food samples rather than technical mixtures. This makes direct comparison with the other studies less straightforward, because additional sources of variability, including sample preparation, matrix effects, and possible laboratory background contamination, are introduced. As a result, a greater degree of mismatch between homologue profiles would be expected.

Because PCAs do not ionize efficiently in either ESI or APCI without suitable additives, LC–MS methods commonly use mobile-phase or post-column additives to promote adduct formation and improve sensitivity toward the desired ions, such as [M + Cl]^−^ or [M + CH_3_COOH-H]^−^. The effects of such additives on ionization are discussed further.

### Additives used to promote PCA ionization

Different additives, such as ammonium chloride (NH_4_Cl), ammonium acetate (NH_4_CH_3_COO), and dichloromethane (DCM), are commonly introduced. These additives promote the formation of specific adduct ions, thereby enhancing both the sensitivity and selectivity of LC–MS methods for PCAs.

The most widely applied approach is using chlorine-containing additives, which promote the formation of chloride adducts ([M + Cl]^−^). Two additives are commonly employed: NH_4_Cl (McGrath et al. 2022a; Kutarna et al. 2023), typically added at low concentrations to the mobile phase (e.g., 25 µM), and dichloromethane (DCM) (Mézière et al. 2020a, b), which can be introduced either directly into the mobile phase or post-column. Zheng et al. (2019) compared the performance of ESI–MS and APCI-MS, showing that ESI consistently produced higher responses than APCI, regardless of whether ammonium chloride (0.5 mM) or dichloromethane (10% v/v) was used as an additive. As discussed in previous section, [M-H]^−^ formation is often observed regardless of the additive used. It has been shown that deprotonated ions of PCAs result from the dissociation of anionic adducts such as [M + Cl]^−^, which then lose HCl, forming [M-H]^−^ species (Zheng et al. 2019).

Recently, other chlorine-containing additives have been tested and successfully applied. Sun et al. (2022) developed a non-target screening method for LC-ESI-Orbitrap MS analysis of different polyhalogenated alkyl compounds, including PCAs, using 10 µM tetraphenylphosphonium chloride (Ph_4_PCl). The study showed that the use of Ph_4_PCl substantially improved PCA response, yielding instrumental detection limits of 0.01–0.03 pg/µL for PCAs-C_10–13_, 0.02–0.06 pg/µL for PCAs-C_14–17_, and 0.04–0.05 pg/µL for PCAs-C_18–25_. Improved sensitivity was also observed for other polyhalogenated organic compounds, including dechloranes, legacy POPs, and brominated flame retardants. Meanwhile, Huang et al. (2021) used tetramethylammonium chloride ((CH_3_)_4_NCl) at 50 µM to improve PCA ionization in ESI source. The study demonstrated that using (CH_3_)_4_NCl as a mobile phase additive greatly improved chloride adduct formation and ionization efficiency of PCAs compared with traditional modifiers such as acetic acid/ammonium acetate or DCM. With (CH_3_)_4_NCl, [M + Cl]^−^ ions were the dominant species, giving several-fold higher responses and cleaner MS spectra. Unlike DCM, which enhances ionization through Cl^−^ release at high concentrations, (CH_3_)_4_NCl was shown to dissociate directly into (CH3)_4_N^+^ and Cl^−^ in the spray process, providing higher sensitivity at much lower additive levels (Huang et al. 2021).

Another widely used option is to use ammonium acetate to promote the formation of acetate adduct ions ([M + CH_3_COOH-H]^−^). Similarly to the generation of chloride adducts, deprotonated ions are formed alongside the desired acetate adducts, particularly noticeable for homologues with high chlorine content (Matsukami and Kajiwara 2019; Matsukami et al. 2020; Zhou et al. 2020). Acetate forms weaker adducts than chloride, as the relative intensity of deprotonated ions to desired adducts is higher when using an acetate additive (Cariou et al. 2016; Matsukami and Kajiwara 2019; Mézière et al. 2020a). Yet, the chloride adducts slightly increase the complexity of the PCA mass spectrum (more Cl atoms lead to extra isotopologue signals). This can increase isobaric interferences in low-resolution MS/MS. Acetate adducts, although less stable, do not increase spectral complexity through additional chlorine-derived isotopologue signals. Besides, fragmentation of chloride adducts and deprotonated ions is nearly identical (loss of HCl), while acetate adducts first lose acetic acid and only then HCl, producing a more characteristic MS/MS spectrum. This makes them useful for PCA determination by LC–MS/MS (Perkons et al. 2025).

Finally, bromide adduct formation has also been explored using bromoform (CHBr_3_). One study (Yuan et al. 2018) using direct-injection APCI-MS achieved near-exclusive formation of stable bromide adducts ([M + Br]^−^). Similarly, Parvizian et al. (2024) promoted bromide-adduct formation by adding 2 µL of bromoform directly to each sample immediately before analysis, such that PCAs were detected as [M + Br]^−^ ions through pre-injection sample spiking rather than by introducing bromoform via the mobile phase or directly before the ionization source. Although bromide adducts appear to provide cleaner spectra by avoiding interferences related to chlorine losses, the use of bromoform has raised concerns because of its higher toxicity compared with chlorinated additives (Yuan et al. 2018).

### Dependence of instrumental response on PCA composition

A well-known challenge in the quantitative analysis of PCAs by LC–MS is that instrumental response is not constant across different PCA homologue groups, but depends strongly on both carbon chain length and chlorination degree. For example, in four studies (Li et al. 2017; Wu et al. 2019; Huang et al. 2021; Amoura et al. 2025) in which PCA quantification was performed using the same commercial standards but different LC-HRMS configurations and ionization additives, the total instrumental response of PCAs-C_10–13_ increased approximately three- to fourfold between SCCP standards containing 51.5% and 63% Cl. Similarly, for PCAs-C_14–17_, the response increased approximately three- to fivefold between MCCP standards containing 42% and 57% Cl. The only exception among these studies was reported by Li et al. (2017), who observed a 40-fold lower response for the 42% Cl MCCP mixture than for the 57% Cl mixture. The more limited data available for PCAs-C_18–23_ likewise indicated a four- to sevenfold increase in response between standard mixtures containing 36% and 49% Cl.

It should be noted, however, that these four studies covered only relatively narrow ranges of chlorine content and did not include highly chlorinated PCA standards. Bogdal et al. (2015), using direct liquid injection APCI-ToF–MS at substantially lower flow rates, observed an approximately 50-fold difference in response between the least- and most-chlorinated PCAs-C_10–13_ mixtures, containing 49% and 70% Cl, respectively. Their results also showed that the increase in response does not continue indefinitely: an LCCP mixture containing 70% Cl produced an instrumental response approximately 30% lower than that of the corresponding mixture containing 49% Cl. This is consistent with homologue-level observations from Yuan et al. (2017), who found that LC–MS response tended to increase up to an intermediate chlorination level, approximately C_n_Cl_10_, but decreased once the number of chlorine atoms exceeded ten.

Overall, PCAs-C_10–13_ tend to produce lower responses than PCAs-C_14–17_ and PCAs-C_18+_, while more highly chlorinated homologues generally exhibit stronger instrumental responses up to a certain limit. These differences must therefore be considered during quantification, because standards with different chlorine contents can yield substantially different response factors. In addition, instrumental response may also depend on congener structure. Fernandes et al. (2022) reported stronger responses for congeners with chlorine atoms distributed along the carbon chain, whereas low chlorination and extensive terminal substitution were associated with poorer response. This structural dependence must be considered when evaluating the suitability of individual congeners as quantification standards for broader PCA mixtures.

## Spectral complexity and interferences in PCA mass spectra

Regardless of the ionization strategy used, LC–MS analysis of PCAs is complicated by considerable spectral complexity. PCA ions form dense isotopic patterns, may appear as several competing ion species, and can be accompanied by signals from co-occurring chlorinated compounds. These factors increase the likelihood of overlap and analytical interference.

First, like other polychlorinated compounds, PCAs generate multiple high-intensity isotopologue signals for each molecular formula. While typical organic molecules composed of C, H, N, and O produce only two to three isotopologues above 10% relative abundance, PCAs yield several due to the presence of two stable chlorine isotopes, ^35^Cl and ^37^Cl, that are both sufficiently abundant to generate multiple intense isotopologue signals. For example, the chloride adduct of C_10_H_18_Cl_4_ produces five isotopologues above this threshold, while C_17_H_28_Cl_8_ produces nine. As a result, PCA ions yield dense isotopic envelopes that can overlap when multiple homologue groups co-elute, complicating ion assignment.

Secondly, in addition to the commonly observed co-formation of deprotonated ions, PCA ionization may also give rise to off-target adducts caused by residual additives remaining in the LC system. For example, Amoura et al. (2024) observed formate adducts ([M + HCOOH-H]^−^) of PCAs with both MeOH and ACN as mobile phases and suggested that these ions originated from residual formic acid remaining in the LC system. Similarly, acetate adducts could also be formed if residual acetate is present from previous use of acetate-containing mobile phases. Such carryover of additives may complicate spectral interpretation and should be considered when assigning observed ion species.

Another important source of spectral interference arises from PCA transformation products that may either form during analysis or already be present in the sample. Schinkel et al. (2017) highlighted possible interferences from thermal degradation products, such as chlorinated olefins and diolefins (COs and CdiOs), also known as chlorinated alkenes (CP-enes), which could form during ionization and cause interferences in mass spectra. The authors investigated the formation of COs and CdiOs from a temperature-exposed mixture of PCAs-C_13_. Using chloride adducts led to an apparent increase in chlorinated olefins, since [M-H]^−^ ions of PCAs overlap with [M + Cl]^−^ adducts of the olefin products. As a result, elevated olefin levels were detected even in unexposed mixtures with chloride enhancement, while acetate adducts showed minimal interference. In exposed samples, both ESI and APCI gave similar PCA transformation product profiles, indicating that either ionization source can be used to study COs and CdiOs (Schinkel et al. 2017, 2018b). Meanwhile, Li et al. (2018) demonstrated that CP-enes are present in technical CP mixtures and that their relative abundance is particularly notable in longer-chain products. The authors further argued that these compounds are not primarily formed during ionization, because CP-enes were detected in commercial technical mixtures but were absent in commercial standard mixtures, indicating that they originate from the manufacturing process rather than from in-source decomposition. CP-enes may also be relevant in samples subjected to acidic cleanup procedures. Godéré et al. (2025) and (Hutter et al. 2025) detected mono- and diolefinic PCAs using a sample preparation protocol involving acidified silica, indicating that CP-enes remain detectable after this treatment. Their potential interference should therefore not be assumed to be limited to raw technical mixtures or materials without extensive cleanup. Recently, Godéré et al. (2025) highlighted a specific group of compounds—vinyl chloride oligomers (VCOs). VCOs are often present in different polyvinyl chloride (PVC) items and products and pose a risk for leaching into the environment or food products. Although the toxicological significance of VCOs and their contribution to the adverse effects attributed to PCAs remain unclear, they are relevant from an analytical perspective because they fall within the compositional space of PCAs. Depending on the analytical objective, VCOs may therefore contribute to the measured PCA signal or complicate the interpretation of patterns attributed specifically to CP technical mixtures. Godéré et al. (2025) proposed a methodology to distinguish PCA signals arising from CP technical mixtures from those arising from VCOs by pattern-level and chromatographic criteria: VCOs form limited, polymer-repeat series (based on the C2H3Cl unit) with a comparatively restricted set of CnClm groups and a fixed chlorine number for a given chain length (C_2n_H_3n+1_Cl_n+1_), whereas PCAs from CP technical mixtures show a much broader spread of PCAs-C_n_Cl_m_ groups. In addition, the latter produce broad, unresolved chromatographic humps (high isomer diversity) while VCOs show narrower peaks.

**Fig. 3:**
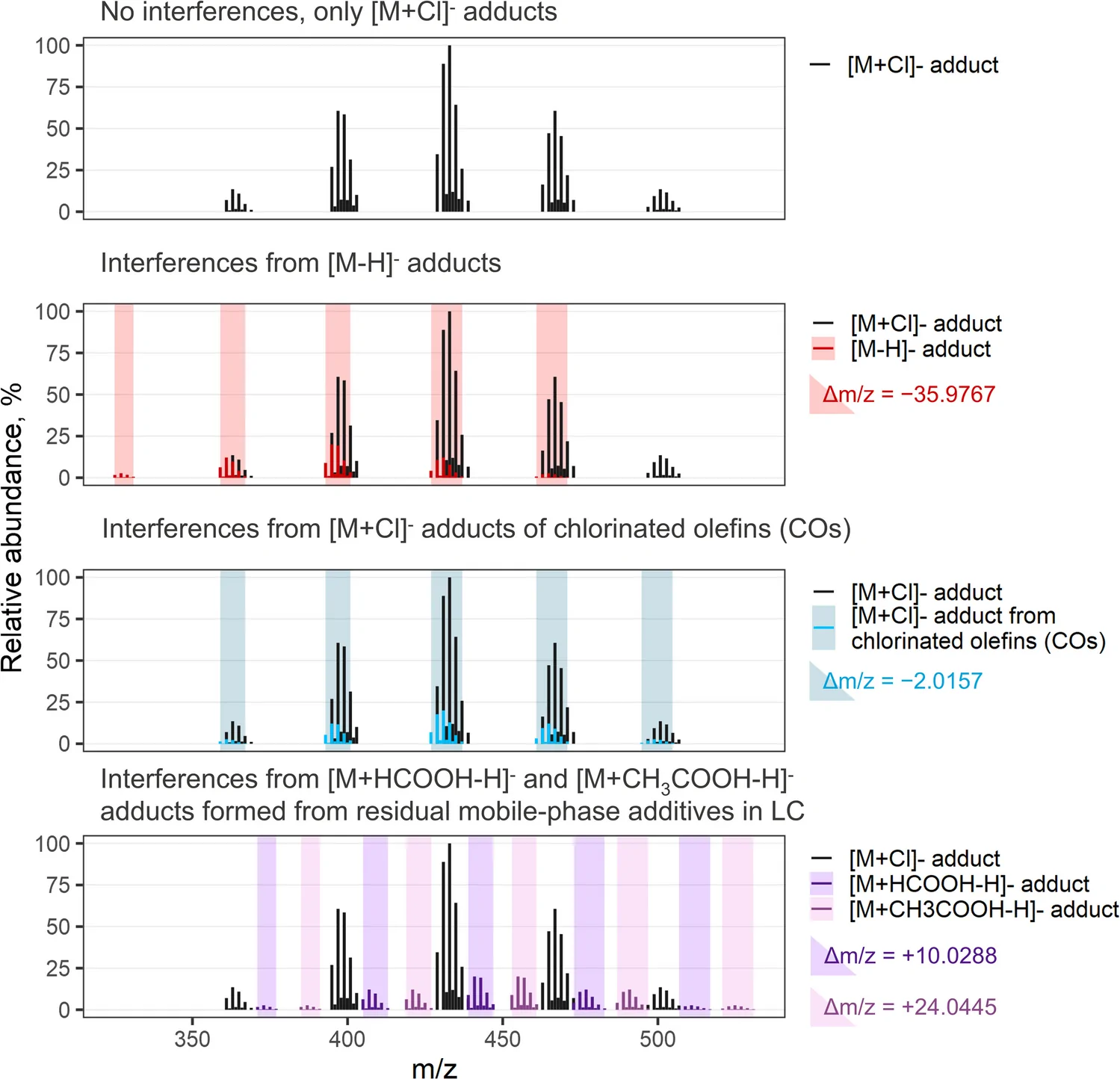
Illustrative mass spectra of PCAs-C_11_Cl_5–9_ chloride adducts and common interferences. The spectra were calculated in R using the enviPat package (Loos et al. 2015). The relative response contributions of the PCAs-C_11_Cl_5–9_ homologue groups were assumed to follow a normal distribution (PCAs-C_11_Cl_5_—5.45%, PCAs-C_11_Cl_6_—24.42%, PCAs-C_11_Cl_7_—40.26%, PCAs-C_11_Cl_8_—24.42%, PCAs-C_11_Cl_9_—5.45%) and all interfering ions were set to 20% of the intensity of the corresponding chloride-adduct signal. The right-hand panel shows the Δm/z of each interference relative to the main [M + Cl]^−^ ion

Figure 3 illustrates the spectral complexity of PCAs using a hypothetical example of PCAs-C_11_Cl_5–9_. In addition to the [M + Cl]^−^ adduct isotopic envelopes, the figure includes several common interfering species mentioned in the previous paragraph: [M-H]^−^ ions, [M + Cl]^−^ ions from chlorinated olefins, and [M + HCOOH-H]^−^, and [M + CH3COOH-H]^−^ adducts formed from residual mobile-phase additives in the LC system. The right-hand side of Fig. 3 shows the Δm/z values of each interference relative to the main [M + Cl]^−^ ion.

Even in the simplified example shown in Fig. 3, where only one single-chain PCA mixture is depicted, interferences originating from higher abundance PCAs can substantially affect low-abundance PCA homologue groups. In real measurements, where dozens of PCAs with different chain lengths may co-elute, the effect of these interferences on the overall PCA profile, and consequently on quantification, is likely to be even more pronounced. Since complete chromatographic separation of PCA homologues is not feasible, high resolving power becomes critical for mitigating these overlaps.

## Mass spectrometric detection strategies for PCAs

To minimize overlap-related interferences discussed in the previous section, PCA analysis generally benefits from instruments with high resolving power, such as Orbitrap MS and higher-end ToF systems. Mézière et al. (2020a) assessed theoretical mass-overlap risks in PCA analysis by considering both overlaps among diagnostic chloride-adduct ions and additional interferences arising from isotopologues of off-target ion species (i.e., deprotonated and acetate adduct ions). The authors showed that, although unresolved overlaps remain, particularly for LCCPs, Orbitrap MS at a resolution of 140,000 was sufficient to resolve most key overlaps, including the separation of [M + Cl]^−^ signals from interfering [M-H]^−^ ions across much of the relevant m/z range.

**Fig. 4:**
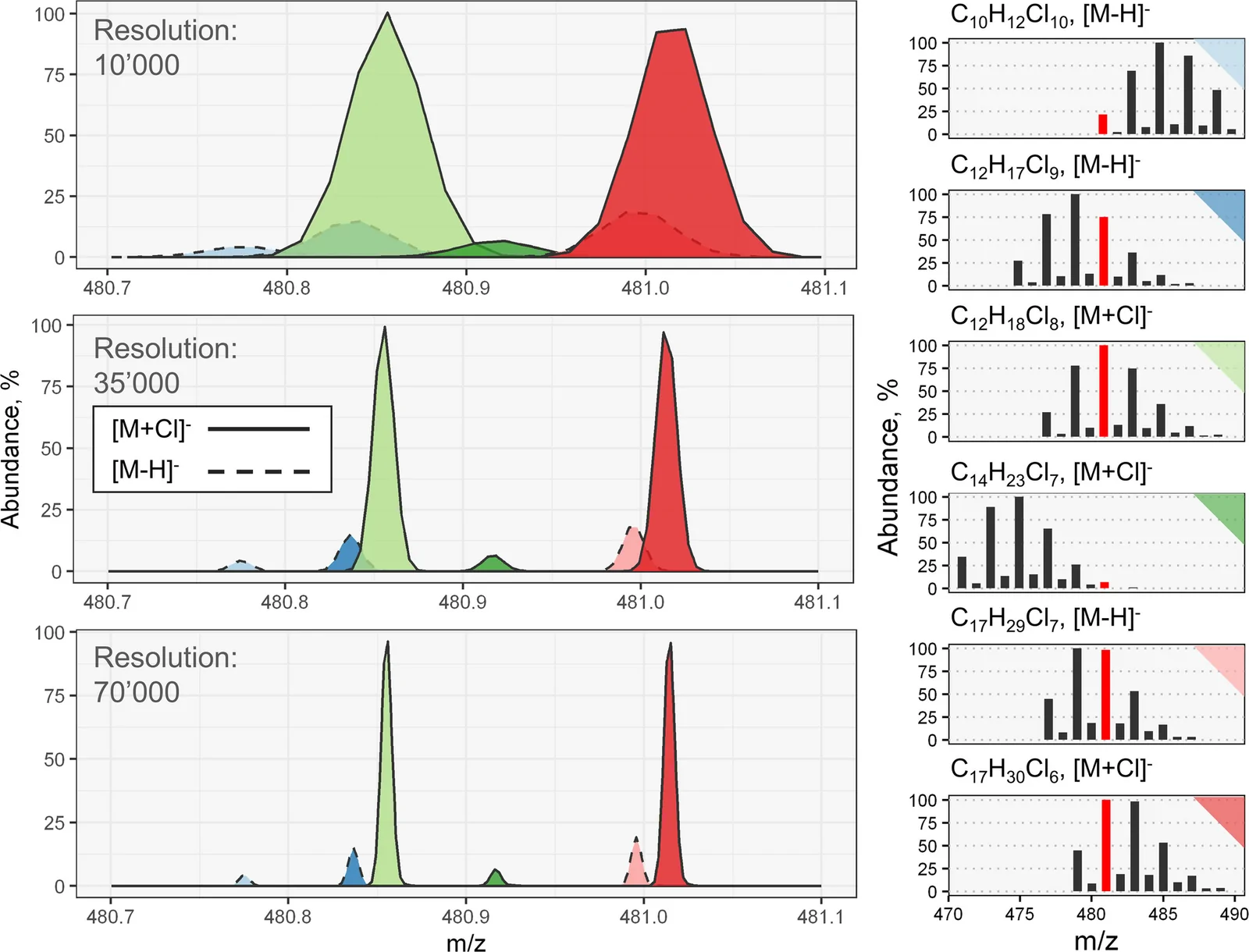
Influence of mass resolving power on the separation of overlapping PCA [M + Cl]^−^ and [M-H]^−^ ions. The simulated spectra include all PCAs-C_10–17_ homologue groups producing signals within the m/z range 480.7–481.1. Resolving powers of 10,000, 35,000, and 70,000 were used to represent lower-resolution ToF, higher-resolution ToF, and Orbitrap MS conditions, respectively

To illustrate this, Fig. 4 depicts a narrow segment (m/z 480.7–481.1) of a theoretical mass spectrum of six PCA homologue groups, in which chloride adducts are the dominant ionic species and deprotonated ions contribute 20% of the intensity of the corresponding chloride adducts. The profile spectra shown in this figure were calculated in R using the enviPat package (Loos et al. 2015). All resolving power values in Fig. 4 are defined using the FWHM criterion. Each resolution setting is intended to mimic instrumental conditions commonly used in HRMS-based methods reported in the literature: 10,000 for lower-resolution ToF instruments, 35,000 for higher-resolution ToF instruments, and 70,000 for Orbitrap MS. The Orbitrap MS setting was chosen to illustrate that the relevant interference can already be resolved at a resolving power of 70,000 (FWHM), rather than to reproduce the maximum resolving power typically available on Orbitrap instruments, which is commonly 120,000 or 140,000 (FWHM), depending on the instrument model. Within this m/z range, the two most abundant PCA chloride-adduct isotopologue signals, [C_12_H_18_Cl_8_ + Cl]^−^ and [C_17_H_30_Cl_6_ + Cl]^−^, are present and remain well separated even at modest resolution. However, both signals may be compromised by interference from [M-H]^−^ species originating from PCAs with the same chain length but one additional chlorine atom. The Δm/z between [PCAs-C_n_Cl_m_ + Cl]^−^ and [PCAs-C_n_Cl_m+1_-H]^−^ is only 0.019, and full separation is achieved only at the highest resolution setting. At resolutions of 10,000 and 35,000, the deprotonated interference may shift the apparent mass of the parent signal slightly toward lower m/z values. This may cause the expected peak to disappear or become abruptly truncated during data processing and peak integration if the mass shift moves it outside the applied mass tolerance window, for example ± 5 or ± 10 ppm. For instruments that cannot sustain sufficiently high resolving power, one way to detect such interferences would be to use wider mass tolerance windows when constructing extracted ion chromatograms and to monitor the mass drift of the expected peak. For peaks showing substantial mass drift, a deconvolution procedure could then be applied during data post-processing to reduce the negative effect of interferences (Schinkel et al. 2017). For example, Mézière et al. (2020b) observed clear differences between LC-ESI-ToF–MS and LC-ESI-Orbitrap-MS. Although both approaches enabled determination of PCA homologue groups, the ToF-based method required deconvolution to address mass overlaps and interferences, whereas the Orbitrap-based method supported a broader range of quantification approaches without this additional step. The authors also reported differences in estimated chlorine content and homologue profiles, likely reflecting differences in ionization conditions and instrumental characteristics. Although deconvolution can improve selectivity, it also increases data-processing complexity and depends strongly on the quality and suitability of the standards used.

In addition to improved selectivity, HRMS offers the further advantage of generating full-scan data for broader qualitative analysis. Such data can support the evaluation of PCA homologue groups outside the original analytical scope, as well as the detection of transformation products such as CP-enes and other halogenated pollutants. The main advantage of HRMS is that it enables PCA quantification while simultaneously generating comprehensive full-scan data. This enables supplementary qualitative analysis of PCA homologue groups outside the initial scope, as well as the identification of PCA transformation products (e.g., CP-enes) and other halogenated pollutants. Moreover, reliable HRMS instrumentation offers possibilities for direct injection analysis methods, which are a time-saving alternative to conventional analysis methods, as they do not involve chromatographic separation (Bogdal et al. 2015). Without chromatographic separation, the influence of interfering ions is expected to be more prominent. Systems with ultra-high resolving power, such as Orbitrap MS or Fourier-transform ion cyclotron resonance MS (FT-ICR-MS), can distinguish homologue ions from interferences without any separation. Schinkel et al. (2018b) demonstrated how direct injection into an Orbitrap system with a resolution power of ~ 100,000 can distinguish between ions and successfully analyze PCAs. However, if the instrument's resolving power is insufficient (e.g., 10,000), additional pattern deconvolution is needed to prevent interfering factors from affecting quantification results (Bogdal et al. 2015; Schinkel et al. 2017). For example, Schinkel et al. (2017) demonstrated that overlapping PCA and CP-ene isotope clusters can be mathematically deconvoluted from direct-injection APCI-qToF-MS data, enabling analysis with relatively lower resolution power (~ 10,000) than other HRMS systems. Direct-injection methods may also offer greater flexibility in the choice of ionization and detection strategies, because sample introduction is no longer constrained by the requirements of chromatographic separation and LC-compatible conditions. For example, Masucci et al. (2024) evaluated atmospheric-plasma-source absorption mode using Orbitrap MS. They relied on liquid sampling atmospheric pressure glow discharge (LS-APGD) ionization in combination with an Orbitrap system. Additionally, they enhanced resolution and sensitivity with the absorption mode Fourier transformation spectral representation, resulting in the resolution of interferences. The employed LS-APGD ionization could also be a novel sample introduction method for PCAs, with the potential of a new nitrate adduct [M + NO_3_]^−^ to be explored further.

Because low-resolution mass spectrometry (LRMS) cannot resolve ions of similar mass, its use in LC–MS analysis of PCAs has been relatively limited. By contrast, LRMS-based GC methods have been more widely applied, as the selectivity of negative chemical ionization reduces the formation and detection of interfering ions, thereby reducing many matrix-related interferences. However, in-source fragmentation of PCAs can still generate additional ions that cause mass interferences (Yuan et al. 2016). Recently, studies have investigated the use of LC–MS/MS to analyze PCAs. Matsukami et al. (2020) developed an LC–MS/MS method to determine the content of PCAs-C_10–13_ in mixed plastic waste in Japan. They relied on acetate adducts of the two most intense isotopologues for each homologue as precursors and acetate and respective homologue ions as product ions. Possibly, their choice was based on the fact that the use of acetate could lower interferences. Acetate provides less complicated mass spectra, and product acetate ions are exclusive to fragmentation of the adducts, unlike chloride adducts, where chloride ions could come from adduct fragmentation and HCl loss from PCA molecules. This method has since been applied to analyze different technical mixtures, and plastic and rubber consumer products in Japan, Belgium, Vietnam, and South Africa (McGrath et al. 2021b; Guida et al. 2022, 2023; Tran et al. 2025; Nevondo et al. 2025). Meanwhile, Perkons et al. (2025) implemented a comparable acetate-adduct MS/MS approach for PCAs-C_10–17_ and demonstrated that it can be used for trace-level quantification in food. Additionally, in an interlaboratory study (Hanari and Nakano 2022) tandem mass spectrometry performed comparably to HRMS methods (ToF), reinforcing that MS/MS strategies are efficient and reliable. However, a practical limitation of LC–MS/MS for PCA analysis is not only MS resolution, but also the large number of multiple reaction monitoring (MRM) transitions required. Because PCA peaks are typically broad and prone to co-elution, multiple MRM transitions often need to be monitored simultaneously within the same retention time window. During the most “crowded” parts of the chromatogram, this may effectively require monitoring the full set of MRM transitions concurrently. Even a relatively narrow scope such as PCAs-C_10–13_Cl_5–8_ requires 32 transitions, whereas expanding the method to PCAs-C_10–20_Cl_4–10_ would increase this number to more than 100. This substantially lengthens the cycle time and reduces the number of data points acquired across each chromatographic peak, which may negatively affect both peak shape and method accuracy. Alternatively, the MRM transitions can be divided across multiple injections, which preserves a sufficiently short cycle time but increases analysis time.

## Conclusions

Liquid chromatographic separation remains an important but inherently limited part of LC–MS analysis of PCAs. Complete separation of PCA homologue groups, let alone individual congeners, is not currently achievable because of the extreme diversity of these mixtures. Still, the reviewed studies show that selection of adequate LC parameters is not insignificant: stationary-phase chemistry, mobile-phase composition, and gradient program all influence PCA retention. Among the tested phases, CN, Phenyl-Hexyl, and C30 columns appear particularly promising for improving chain-length separation, but substantial overlap between homologue groups remains unavoidable. As a result, LC optimization should be viewed as a way to reduce, rather than eliminate, the burden placed on MS detection.

The choice of additive is one of the main factors determining ionization efficiency, spectral complexity, and ultimately method selectivity. Chlorine-containing additives remain the dominant strategy because they promote formation of [M + Cl]^−^ ions and generally provide the highest sensitivity. More recently introduced additives, such as Ph_4_PCl and (CH_3_)_4_NCl, show that meaningful gains in sensitivity and signal cleanliness are still possible, whereas bromide-adduct strategies illustrate that alternative adduct chemistries may also be explored. At the same time, acetate-based approaches retain clear value, particularly for LC–MS/MS workflows, because they can produce more characteristic fragmentation pathways. Overall, additive selection should be treated as a central method design choice that determines which ions are formed, how complex the spectra become, and which detection strategy is most suitable.

A recurring conclusion of this review is that PCA mass spectra are intrinsically complex and prone to overlap. This complexity arises not only from dense chlorine-driven isotopic envelopes, but also from co-formation of deprotonated ions, residual-adduct formation, and the presence of PCA-related compounds such as CP-enes. Because complete chromatographic separation is not feasible, high resolving power becomes critical for controlling these overlaps and preserving method reliability. HRMS therefore remains the most capable detection strategy, particularly because it combines improved selectivity with full-scan data that can support broader qualitative analysis. By contrast, LC–MS/MS can be used for quantitative analysis of more narrowly defined PCA ranges, where qualitative PCA profile characterization is of secondary importance. Direct-injection approaches further illustrate both the promise and the limits of modern HRMS-based PCA analysis. By removing chromatographic constraints, they offer faster analysis and greater flexibility in sample introduction and ionization design, but they also introduce the full extent of unresolved spectral complexity. For this reason, such methods are most convincing when coupled to ultra-high-resolution instruments or robust deconvolution strategies.

Regardless of the LC–MS configuration used, PCA response factors vary across homologue groups and congeners. This variability is influenced by carbon-chain length, chlorination degree, and congener structure. Reliable quantification therefore requires standards that adequately represent the chain-length and chlorination profile of the analysed sample. Taken together, the reviewed literature suggests that no universal LC–MS method for PCAs yet exists. Instead, the most suitable approach depends on the analytical purpose, including the PCA range of interest, the required level of selectivity, and whether broad PCA profiling or targeted quantification for sum PCAs is the primary goal.

## Data Availability

All data analyzed are included in this published article.
